# Metabolic active peritoneal sites affect tumor debulking in ovarian and peritoneal cancers

**DOI:** 10.1186/s13048-020-00662-3

**Published:** 2020-05-20

**Authors:** Tung Tung Tsoi, Keith W. H. Chiu, M. Y. Chu, Hextan Y. S. Ngan, Elaine Y. P. Lee

**Affiliations:** 1 Department of Diagnostic Radiology, Queen Mary Hospital, The University of Hong Kong, Room 406, Block K, Queen Mary Hospital, Pokfulam Road, Hong Kong, Hong Kong; 2 Department of Obstetrics and Gynaecology, Queen Mary Hospital, The University of Hong Kong, 6/F, Professorial Block, Queen Mary Hospital, Pokfulam Road, Hong Kong, Hong Kong

**Keywords:** ^18^F-FDG PET/CT, Ovarian and peritoneal cancers, Tumor debulking, Peritoneal carcinomatosis

## Abstract

**Rationale and objectives:**

To evaluate the impact of metabolic parameters in the peritoneal cavity on the likelihood of achieving complete tumor debulking in patients with ovarian and peritoneal cancers.

**Materials and methods:**

Forty-nine patients with ovarian and peritoneal cancers were included, who underwent pre-operative ^18^F-fluorodeoxyglucose Positron Emission Tomography/Computed Tomography (^18^F-FDG PET/CT). The immediate surgical outcome was dichotomized into complete and incomplete tumor debulking. ^18^F-FDG PET/CT was qualitatively and quantitatively assessed by scrutinizing 15 anatomical sites for the presence of peritoneal carcinomatosis (PC). Patient-based and site-based diagnostic characteristics were described. Metabolic parameters (SUVmax, metabolic tumor volume and total lesion glycolysis) and the number of ^18^F-FDG avid peritoneal sites were evaluated between the two groups. Receiver operating curve (ROC) analysis was performed to determine the optimal cut-off value in predicting incomplete tumor debulking.

**Results:**

Twenty-seven out of the 49 patients had PC and 11 had incomplete debulking. Patient-based and site-based accuracies for detection of PC were 87.8 and 97.6%, respectively. The number of ^18^F-FDG avid peritoneal sites was significantly different between complete and incomplete debulking groups (0.6 ± 0.8 versus 2.3 ± 1.7 sites respectively, *p* = 0.001), and the only independent significant risk factor among other metabolic parameters tested (odd ratio = 2.983, 95% CI 1.104–8.062) for incomplete tumor debulking with an optimal cut-off value of ≥4 (AUC = 0.816).

**Conclusion:**

The number of ^18^F-FDG avid peritoneal sites increased the risk of incomplete tumor debulking after surgery and potentially useful in assisting treatment stratification in patients with ovarian and peritoneal cancers.

## Introduction

Clinical symptoms of ovarian and peritoneal cancers are not apparent until the diseases reach advanced stages and thus hamper early detection [1]. The survival rate of patients with early stage disease is favorable at approximately 90%; however, most cases present late when symptoms become significant [2] with survival dropping to 20 to 30% in advanced disease (International Federation of Gynecology and Obstetrics, FIGO stage III & IV) [1].

Upfront debulking surgery (UDS) followed by chemotherapy is the standard treatment for ovarian and peritoneal cancers. UDS aims at achieving complete tumor debulking in order to be left with no residual disease as this has the most impact on survival outcome [3]. Sometimes, this will require ultra-radical surgical procedures that include maximal stripping of diseased peritoneum and resection of multiple visceral organs to achieve this [1].

Peritoneal carcinomatosis (PC) is a common pathway of tumor dissemination in patients with ovarian and peritoneal cancers [4]. PC is a negative predictor of achieving complete tumor debulking, in which the success rate decreased significantly to 26% compared to 76% in patients without PC [5].

Neoadjuvant chemotherapy (NAC) followed by interval debulking surgery (IDS) provides an alternative treatment option for patients in whom complete UDS cannot be achieved [6]. Standard surgical procedures for both UDS and IDS consist of total abdominal hysterectomy, bilateral salpingo-oophorectomy, omentectomy, and maximum debulking of metastatic tumors. Systematic pelvic lymphadenectomy and/or paraaortic lymphadenectomy could be performed in selected patients. Non-inferiority of this alternative approach (NAC followed by IDS) was subsequently shown in randomised controlled trials with no significant difference in overall survival and in the rate of complete debulking [7–9]. However, there is concern of NAC-induced fibrosis and adhesions that could impact on the perioperative visual assessment of the abdominopelvic cavity, higher risk of platinum resistance in disease recurrence and potentially missing the opportunity for disease debulking in patients refractory to NAC.

Therefore, it is important to select suitable patients for UDS and we are in need of an assessment tool that allow accurate disease evaluation in terms of disease resectability and likelihood of achieving complete tumor debulking.

Previous studies had shown that ^18^F-fluorodeoxyglucose Positron Emission Tomography/Computed Tomography (^18^F-FDG PET/CT) was promising in PC detection [10, 11]. In this study, we aimed to evaluate the impact of metabolic parameters in the peritoneum on the likelihood of achieving complete tumor debulking in patients with ovarian and peritoneal cancers with a secondary aim of evaluating the diagnostic characteristics of ^18^F-FDG PET/CT in the detection of PC.

## Materials and methods

This retrospective study was reviewed and approved by local institutional ethics review board. Written consent was waived. The local database was searched with the following inclusion criteria: i) histological confirmed ovarian or peritoneal cancer (primary and recurrent disease); ii) patients underwent debulking surgery and iii) ^18^F-FDG PET/CT performed prior to debulking surgery. For those with recurrent disease, disease-free and treatment-free period should be at least 12 months from the last treatment cycle. Exclusion criteria were: i) history of previous cancers, other than ovarian or peritoneal cancer; ii) NAC was administered; iii) interval between ^18^F-FDG PET/CT and debulking surgery was longer than 3 months or iv) artifacts on ^18^F-FDG PET/CT that precluded adequate assessment or quantification. Patient’s demographics were collected and serum CA-125 was recorded at the time of ^18^F-FDG PET/CT.

### Immediate surgical outcome

All surgical procedures were performed by experienced gynecologists specialized in oncologic surgery with more than 10 years’ experience. All operating notes were reported in a standardized manner [12]. Standard surgical debulking procedures were performed, including diaphragmatic stripping or resection, bowel resections, appendicectomy and tumor removal from the urinary bladder flap but there was no splenectomy, hepatectomy or gastrectomy performed in this cohort. Normal looking mesenteric lymph nodes were not removed.

During surgical exploration, visual inspection and palpation of visceral abdominopelvic organs were performed. The location, extent and dimension of visible disease and disease-free site were systematically recorded in the surgical note for each patient. Any resected or biopsied specimens was sent for histopathological examination. Histopathological assessment was evaluated by experienced pathologist with special interest in gynecologic oncology and discussed at the weekly multidisciplinary meetings.

The operating notes and histopathological reports of patients enrolled were reviewed and these were considered as the standard of references. The result of histopathology was regarded as the gold standard if there was a discrepancy between operative finding and histopathology. Therefore, site that was thought to be positive at surgery but proven negative by histology was regarded as negative for malignancy and vice versa.

The abdominopelvic cavity was assessed at the end of debulking surgery and patients were dichotomized into two groups; complete tumor debulking was defined as no residual disease after surgery and incomplete tumor debulking was defined as the presence of residual disease (regardless of size) at the end of surgery.

The presence of PC was further divided into two categories based on size of the PC lesion, subcentimeter lesion that included lesion with less than 1 cm or microscopic malignant disease and lesion ≥1 cm. All lesions were pathologic proven to be malignant.

### Patient preparation, imaging protocol and reconstruction parameters

All patients were required to fast for at least 6 h with their blood glucose level verified to be below 10 mmol/L before administration of ^18^F-FDG. After injection, patients were asked to rest during the uptake phase to avoid elevated uptake of skeletal musculature.

All ^18^F-FDG PET/CT examinations were performed on an integrated PET/CT scanner (Discovery VCT; 64-MDCT, GE Healthcare Bio-Science Corp., Piscataway, NJ, USA). The injected dose of ^18^F-FDG was weight-dependent (298 + 53 MBq). Data acquisition started approximately 1 h after intravenous injection of ^18^F-FDG. CT scan was obtained covering from the skull base to proximal thigh with or without intravenous injection of iodinated contrast, using the following imaging parameters: 120 kVp, 200–400 mA, slice thickness of 2.5 mm, a 50 cm field of view (FOV) with 512 × 512 matrix, rotation of 0.5/s and pitch of 0.984. For contrast-enhanced CT, intravenous iodinated contrast was injected at a rate of 2.5 ml/sec via the antecubital vein. PET was acquired after CT data acquisition with the following parameters: approximately 6 bed positions per patient with 2.5 min per bed position. CT data was used for attenuation correction for PET data reconstruction with an OSEM algorithm using 2 iterations and 14 subsets.

### Image and data analyses

All ^18^F-FDG PET/CT examinations were retrospectively analyzed on dedicated workstation (Advantage Workstation, 4.3, GE healthcare, NJ, USA) by board-certified radiologist (> 10 years’ experience in cross-sectional imaging and > 7 years’ experience in ^18^F-FDG PET/CT) who was blinded to the immediate surgical outcome. Fifteen anatomical sites were scrutinized for the presence of PC: right subphrenic, right subhepatic, gastric serosa, lesser sac, left subphrenic, left perihepatic, right paracolic, left paracolic, POD, bladder flap, mesentery, omentum, large bowel serosa, small bowel serosa and pelvic regions. Abnormal metabolic uptake above background activity with correlated altered anatomical changes on CT in the aforementioned 15 anatomical sites were defined as presence of PC [13]. CT information was co-registered for lesion localization. Intraperitoneal plaque like or nodular soft tissue mass with or without ascites was regarded as suspicious malignant intraperitoneal deposit [4]. Round, “cake-like”, ill-defined and stellate masses in the sites of mesentery and omentum were regarded as malignant involvement [4]. Bowel with wall thickening, nodular or mass-liked infiltration was regarded as bowel serosa infiltration [14]. The number of hypermetabolic sites in the peritoneum was recorded based on the aforementioned 15 sites.

Rectangular 3-dimensional volume of interest (VOI) was inserted on PET images to cover the entire tumor and adjusted to exclude surrounding non-tumor activity to measure standardized uptake value (SUV). SUVmax was defined as the maximum value of SUV within the VOI. Metabolic tumor value (MTV, cm^3^) and total lesion glycolysis (TLG, cm^3^) were calculated based on 45% threshold segmentation [15].

All the suspicious peritoneal uptakes in the abdominopelvic cavity on ^18^F-FDG PET/CT were quantified. The most metabolic active PC lesion was selected for SUVmax quantification for each patient, the total metabolic burden was quantified by summation of the MTV and TLG of all PC lesions for each patient. Furthermore, the number of metabolic active peritoneal sites was recorded based on the 15 anatomical peritoneal sites scrutinized for PC.

### Statistical analysis

Data are expressed as mean value + standard deviation (SD). Normality was tested with the Shapiro-Wilk test. Diagnostic characteristics of ^18^F-FDG PET/CT were calculated and described by accuracy, sensitivity, specificity, positive predictive value (PPV) and negative predictive value (NPV) based on patient-based and lesion-based analyses. The categorical analysis was evaluated by Fisher’s exact test. The differences in the metabolic parameters (SUVmax, MTV, TLG and the number of metabolic active peritoneal sites) and serum CA-125 recorded at the time of ^18^F-FDG PET/CT between complete and incomplete debulking cohorts were analyzed by Mann-Whitney U test. Subsequently, binary logistic regression test was used to test the predictive value of these metabolic parameters on the immediate surgical outcome. For independent significant predictor, the Receiver Operating Characteristic (ROC) curve analysis was used to determine the optimal cut-off values. The cut-off value was chosen by maximizing the specificity and minimizing the number of false positive. Statistics analysis (SPSS, Version 19.0, Chicago, IL, USA) was performed using two-sided *p* values and statistical significance was assumed if *p* < 0.05.

## Results

From April 2008 to January 2019, there were 60 patients who met the inclusion criteria but 11 were excluded as 10 patients received NAC before debulking surgery and 1 patients had long duration (173 days) between the ^18^F-FDG PET/CT and debulking surgery. Therefore, a total of 49 patients were enrolled in this study and the demographics are summarized in Table 1. Epithelial ovarian cancer (EOC, *n* = 43, 87.8%) was the most common type of ovarian cancer in this study; while endometrioid and serous adenocarcinoma were the common subtypes of EOC. Others were germ cell (*n* = 4, 8.2%) and stromal cell (*n* = 2, 4.1%).

**Table 1:** Demographics and immediate surgical outcomes of recruited patients (*n* = 49)

**Age**	49 ± 15
**Status**	
Newly Diagnosed	29
Recurrent	20
**Primary Origin**	
Ovarian Cancer	45
Peritoneal Cancer	4
**Staging**	
Unknown	3
I	15
II	12
III	18
IV	1
**Grade**	
Unknown	17
G1	7
G2	8
G3	17
**Interval between**^**18**^**F-FDG PET/CT and surgery**	19 ± 16 days
**Serum CA-125**	478.2 ± 1028.6 units/mL
**Immediate surgical outcome**	
Complete debulking	38 (77.6%)
Incomplete debulking	11 (22.4%)

**Fig. 1:**
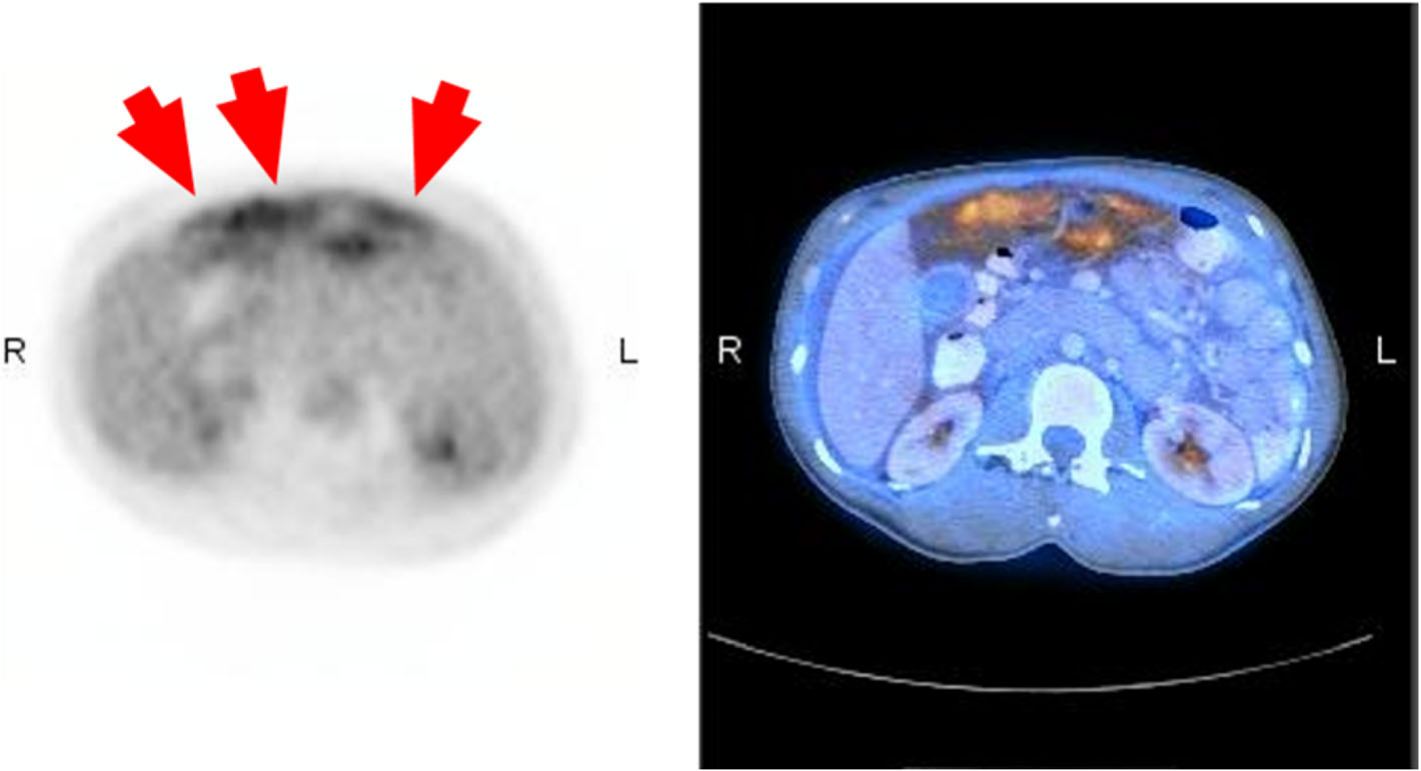
False positive on 18F-FDG PET/CT. Diffuse FDG avid uptake in gastro-colic space with SUVmax (3.2) found in a 38-year-old female patient having recurrent mucinous adenocarcinoma of ovary was confirmed by histology to be fatty tissue with nodular hyperplasia: left image – PET only axial image with red arrows indicating the diffuse uptake; right image – PET/CT fusion axial image showing corresponding uptake in the gastro-colic space

Among 27 patients with histologically confirmed PC, ^18^F-FDG PET/CT correctly identified 25 patients (sensitivity 92.6%, specificity 81.8% and accuracy 87.8%, Table 2). A total of 735 peritoneal sites were analyzed and the site-based diagnostic characteristics of ^18^F-FDG PET/CT are tabulated in Table 2. The detection rates for different peritoneal sites are summarized in Table 3. The frequent sites of peritoneal deposition were in the pelvic region (28.6%), followed by large bowel infiltration (18.4%), omentum (12.2%), the bladder flap (12.2%) and POD (12.2%). ^18^F-FDG PET/CT had low sensitivity at detecting peritoneal metastases involving small bowel serosa (0%), bladder flap (33.3%) and bowel mesentery (40.0%). Three false positive findings were due to nodular mesothelial and histiocytic hyperplasia in the omentum (Fig. 1) and inflammation in the pelvic peritoneum. The remaining false positive case with uptake in the right subphrenic space had no abnormality found at surgery.

**Table 2:** Diagnostic characteristics of ^18^F-FDG PET/CT for peritoneal carcinomatosis

	Patient-based	Site-based ^a^
Total	49	735
Histological positive	27	58
Histological negative	22	677
True positive on ^18^F-FDG PET/CT	25	44
False negative on ^18^F-FDG PET/CT	2	14
False positive on ^18^F-FDG PET/CT	4	4
True negative on ^18^F-FDG PET/CT	18	673
Sensitivity	92.6%	75.9%
Specificity	81.8%	99.4%
PPV	86.2%	91.7%
NPV	90.0%	98.0%
Accuracy	87.8%	97.6%

*Remarks:*^a^*Fifteen anatomical sites were scrutinized for the presence of peritoneal carcinomatosis (right subphrenic, right subhepatic, gastric serosa, lesser sac, left subphrenic, left perihepatic, right paracolic, left paracolic, POD, bladder flap, mesentery, omentum, large bowel serosa, small bowel serosa and pelvis regions)*

**Table 3:** Site-based detection rate of ^18^F-FDG PET/CT for peritoneal carcinomatosis

	Right Subphrenic	Right Subhepatic	Gastric Serosa	Lesser Sac	Left Subphrenic	Left Perihepatic	Right Paracolic	Left Paracolic	POD	Bladder Flap	Mesentery	Omentum	Large Bowel Serosa	Small Bowel Serosa	Pelvis
Total Region	49	49	49	49	49	49	49	49	49	49	49	49	49	49	49
Histological positive	3	2	1	0	1	0	2	2	6	6	5	6	9	1	14
Histological negative	46	47	48	49	48	49	47	47	43	43	44	43	40	48	35
True positive on ^18^F-FDG PET/CT	2	2	1	0	1	0	2	2	3	2	2	5	8	0	14
False negative on ^18^F-FDG PET/CT	1	0	0	0	0	0	0	0	3	4	3	1	1	1	0
False positive on ^18^F-FDG PET/CT	1	0	0	0	0	0	0	0	0	0	0	2	0	0	1
True negative on ^18^F-FDG PET/CT	45	47	48	49	48	49	47	47	43	43	44	41	40	48	34
Sensitivity	66.7%	100.0%	100.0%	–	100.0%	–	100.0%	100.0%	50.0%	33.3%	40.0%	83.3%	88.9%	0.0%	100.0%
Specificity	97.8%	100.0%	100.0%	100.0%	100.0%	100.0%	100.0%	100.0%	100.0%	100.0%	100.0%	95.3%	100.0%	100.0%	97.1%
PPV	66.7%	100.0%	100.0%	–	100.0%	–	100.0%	100.0%	100.0%	100.0%	100.0%	71.4%	100.0%	–	93.3%
NPV	97.8%	100.0%	100.0%	100.0%	100.0%	100.0%	100.0%	100.0%	93.5%	91.5%	93.6%	97.6%	97.6%	98.0%	100.0%
Accuracy	95.9%	100.0%	100.0%	100.0%	100.0%	100.0%	100.0%	100.0%	93.9%	91.8%	93.9%	93.9%	98.0%	98.0%	98.0%

Among 53 PC lesions detected, 9 were subcentimeter and 44 had lesion size ≥1 cm. The largest residual disease had longest dimension of 12 cm, which was a hard tumor in the POD densely adhered to the sigmoid colon and sacrum extending to the left pelvic sidewall. Among the resected specimens (longest dimension 4.7 ± 3.9 cm), the largest lesion was 20 cm. Given this was a retrospective study, information of amount of diseased and normal peritoneal tissue removed was not available. The overall lesion-based detection rate of ^18^F-FDG PET/CT was 83.0% regardless of lesion size but improved to 95.5% (42/44) when only lesions with size ≥1 cm were considered. The detection rate was poor for subcentimeter peritoneal metastases, 22.2% (2/9). There was significant difference in the detection rates between the subcentimeter and ≥ 1 cm peritoneal metastases by ^18^F-FDG PET/CT (*p* < 0.001).

At the completion of debulking surgery, 38 patients (77.6%) achieved complete tumor debulking while 11 cases (22.4%) had incomplete tumor debulking. Patients with positive findings of PC on ^18^F-FDG PET/CT had significant lower rate of achieving complete tumor debulking (19/29, 65.5% versus 19/20, 95.0%) when compared to those without PC detected on ^18^F-FDG PET/CT (*p* = 0.017).

There were significant differences in the metabolic parameters, including SUVmax (*p* = 0.006), MTV (*p* = 0.013), TLG (*p* = 0.007) and number of metabolic active peritoneal sites (*p* = 0.001) between complete and incomplete tumor debulking groups (Table 4). No significant difference was observed in serum CA-125 between two groups (*p* = 0.509) (Table 4).

**Table 4:** Distribution of serum CA-125, SUVmax, MTV, TLG and number of metabolic active peritoneal sites between complete and incomplete tumor debulking cohorts

	Complete tumor debulking cohort	Incomplete tumor debulking cohort	*p*-value*
Serum CA-125 (units/mL)	306.1 ± 422.1	1010.2 ± 1911.9	0.509
SUVmax	2.4 ± 3.4	5.8 ± 3.4	0.006
MTV (cm^3^)	27 ± 49	81 ± 120	0.013
TLG (cm^3^)	62 ± 114	167 ± 190	0.007
Number of metabolic active peritoneal sites	0.6 ± 0.8	2.3 ± 1.7	0.001

Remarks: *p-value obtained by using Mann-Whitney U Test

**Fig. 2:**
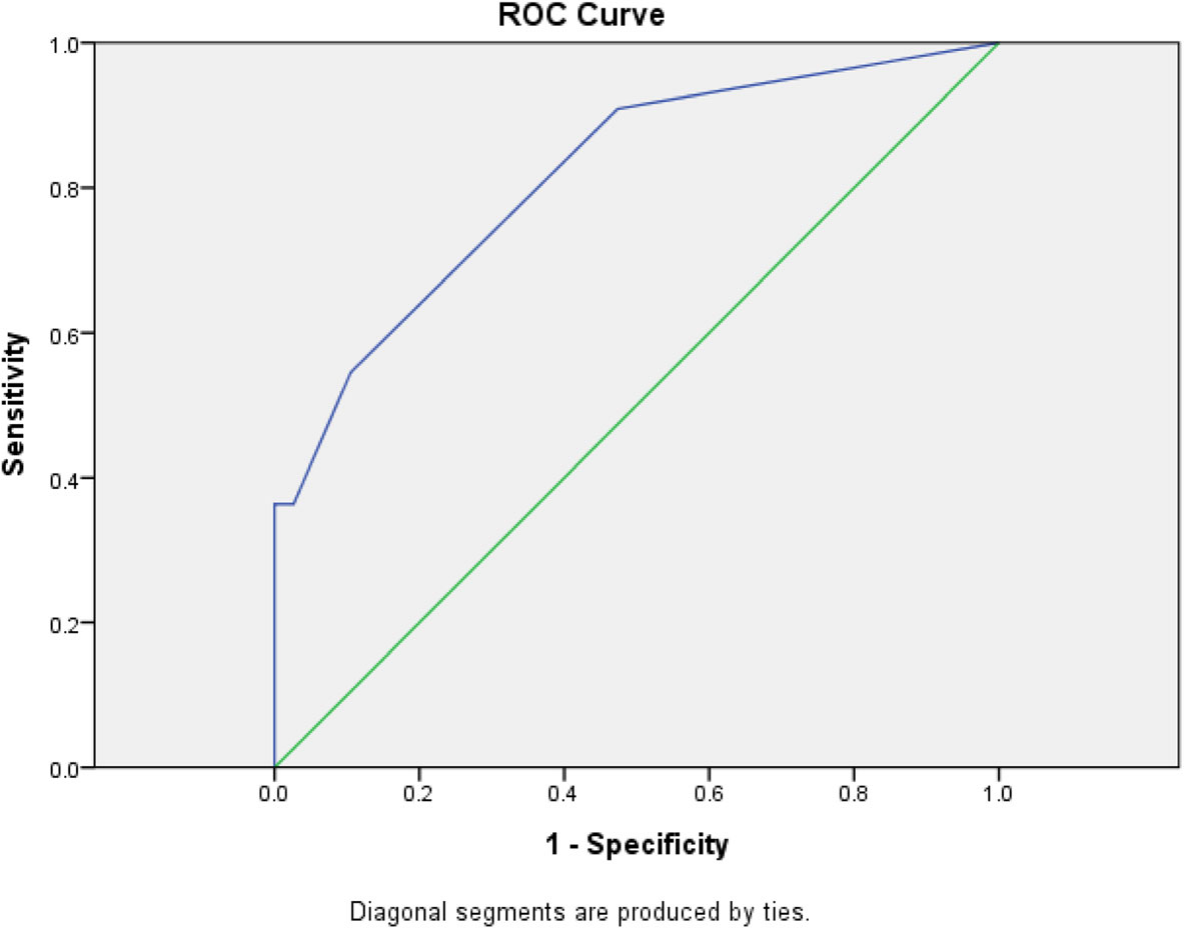
Receiver operating characteristic (ROC) curve of number of positive imaging peritoneal sites detected by ^18^F-FDG PET/CT

In univariate analysis, the presence of PC on ^18^F-FDG PET/CT (*p* = 0.036), SUVmax (*p* = 0.012) and the number of metabolic active peritoneal sites (*p* = 0.003) were significant risk factors for incomplete tumor debulking, whereas other factors (MTV and TLG) were not (*p* > 0.050). In multivariate analysis, the number of metabolic active peritoneal sites was the only significant risk factor for incomplete tumor debulking (*p* = 0.031, standard error = 0.507, odd ratio = 2.983 with 95% CI 1.104 to 8.062). Subsequent ROC curve was generated based on the number of metabolic active peritoneal sites (AUC 0.816, Fig. 2). In order to maximize the specificity, optimal cut-off value of ≥4 metabolic active peritoneal sites was chosen (sensitivity of 36.4%, specificity of 100.0%, PPV of 100.0%, NPV of 84.4%, and accuracy of 85.7%, Table 5).

**Table 5:** Sensitivity, specificity, PPV, NPV, accuracy, unnecessary surgical exploration rate and inappropriately unexplored rate of different cut-off values used in ROC analysis by the number of ^18^F-FDG avid peritoneal sites

No. of ^18^F-FDG avid peritoneal sites ^a^	True Positive	False Positive	False Negative	True Negative	Sensitivity	Specificity	PPV	NPV	Accuracy	Unnecessary surgical exploration ^b^	Inappropriately unexplored ^c^
≥ 0	11	38	0	0	100.0%	0.0%	22.4%	–	22.4%	–	77.6%
≥ 1	10	18	1	20	90.9%	52.6%	35.7%	95.2%	61.2%	4.8%	64.3%
≥ 2	6	4	5	34	54.5%	89.5%	60.0%	87.2%	81.6%	12.8%	40.0%
≥ 3	4	1	7	37	36.4%	97.4%	80.0%	84.1%	83.7%	15.9%	20.0%
≥ 4	**4**	**0**	**7**	**38**	**36.4%**	**100.0%**	**100.0%**	**84.4%**	**85.7%**	**15.6%**	**0.0%**
≥ 5	1	0	10	38	9.1%	100.0%	100.0%	79.2%	79.6%	20.8%	0.0%
≥ 6	0	0	11	38	0.0%	100.0%	–	77.6%	77.6%	22.4%	–

*Remarks:*

^a^*Fifteen anatomical sites were scrutinized for the presence of peritoneal carcinomatosis (right subphrenic, right subhepatic, gastric, lesser sac, left subphrenic, left perihepatic, right paracolic, left paracolic, POD, bladder flap, mesentery, omentum, large bowel serosa, small bowel serosa and pelvis regions)*

^b^*Unnecessary surgical exploration (%): the ratio of patients thought to have resectable disease but who will turn out to have incomplete debulking after surgery; correspond to false negative rate (1 - NPV)*

^c^*Inappropriately unexplored (%): the ratio of patients thought to have unresectable disease but who will turn out to have complete debulking after surgery; correspond to false positive rate (1 - PPV)*

## Discussion

In this study, we demonstrated that the number of metabolic active peritoneal sites impacted on the immediate surgical outcome, in that a higher number of metabolic active peritoneal sites was associated with incomplete tumor debulking in ovarian and peritoneal cancers. Additionally, ^18^F-FDG PET/CT offered high accuracy in PC detection.

^18^F-FDG PET/CT showed good sensitivity of detecting PC on patient-based analysis but only moderate on site-based analysis, implying that it might underestimate the extent and burden of PC. One possible explanation might be due to the site-dependent detection rate of ^18^F-FDG PET/CT. In our study, ^18^F-FDG PET/CT was found to be poor in detecting disease involvement at the small bowel serosa, bladder flap and mesentery. Our findings corroborated with previous study that showed poor detection rate in small bowel serosa infiltration with a sensitivity of only 43% but still superior to contrast enhanced CT at merely 14% [16]. Furthermore, physiologic FDG bowel activity may hamper the detection of serosal disease [17]. Although MRI is superior in detecting serosal disease given the better soft tissue contrast, it suffers from artifacts from breathing or body motion, therefore resulting in overall comparable detection rates between the two imaging modalities [10]. As with bladder flap disease, the physiologic FDG excretion in the urinary bladder will hamper the detection [15].

Besides site-dependence, the detection rate was affected by the size of peritoneal deposit. The detection rate of peritoneal lesions ≥1 cm was superior but drastically decreased when subcentimeter lesions were considered, as shown in our study. Detection of small peritoneal deposits remains a clinical challenge. Both ^18^F-FDG PET/CT and MRI enjoy similar high accuracy in PC detection but underestimate the extent of disease due to decreased sensitivity for subcentimeter lesions [18]. This is conceivable as the spatial resolution of PET is limited by the scintillation properties, detector unit size and electronics hardware. The best image reconstructed by current clinical PET scanners have a full width half maximum (FWHM) of 4 to 7 mm [19]. Aside from the limitation of spatial resolution, partial volume effect or partial volume error might also explain for low detection rate of subcentimeter peritoneal lesions. Study showed that there was significant difference in average SUVmax between lesions smaller and larger than 0.5 cm [20]. This could be one of the reasons accounting for the high incidence of false negative in subcentimeter lesions.

Our results demonstrated that the presence of PC on ^18^F-FDG PET/CT was associated with incomplete tumor debulking as PC is known to be a negative predictor for incomplete debulking [5]. This was further supported by the study by *Risum* et al, which found that the presence of PC detected by ^18^F-FDG PET/CT scan was a significant univariate predictor of incomplete UDS [21].

Other than the presence of PC, SUVmax and the number of metabolic active peritoneal sites were associated with incomplete tumor debulking as more aggressive and extensive disease spread would increase the complexity of the surgical procedures required and decrease the likelihood of achieving complete tumor debulking. However, only the number of metabolic active peritoneal sites was a significant risk factor for incomplete tumor debulking among the metabolic parameters tested. This might suggest that the disease distribution was a more important determinant of surgical success than the metabolic uptake or metabolic tumor burden. Certain critical locations of PC dissemination such as the subphrenic surfaces, lesser sac, porta hepatis and small bowel mesentery hinder the accessibility and resectability at surgery [22]. The assessment based on the number of metabolic active peritoneal sites would be less time-consuming and cumbersome to perform compared to quantification of the tumor burden by placing the VOIs and summation of these values, therefore more conducive to be included as part of the clinical assessment while reporting and interpreting ^18^F-FDG PET/CT.

In this study, the optimal cut-off value was chosen as ≥4 in order to maximize the specificity in reducing the rate of inappropriately unexplored as low as possible. Nevertheless, this could be adjusted to fulfil the local need such as the availability and experience of the surgical team, resource availability and patient’s choice. It could also provide data to advise on the likelihood of successful complete tumor debulking surgery based on the metabolic parameters from ^18^F-FDG PET/CT for better informed consent; vice versa for the oncologists when starting chemotherapy. *Ebina* et al reported that ^18^F-FDG PET/CT had altered 58.4% of treatment planning in recurrent ovarian cancer and the favored treatment choice for debulking surgery was increased from 12 to 35 patients while maintaining a high complete tumor debulking rate (91.4%) [23]. The selection criteria in the study was based on the pattern of ^18^F-FDG uptake (localized, multiple or diffuse) while no quantitative metabolic parameters were included. In contrast, we investigated various quantitative and semi-quantitative metabolic parameters, including SUV, MTV, TLG and the number of metabolic active peritoneal sites, which could offer more objective assessment. Therefore, ^18^F-FDG PET/CT could be beneficial in selection of suitable candidate for UDS and optimizing management plan. A recent study also suggested summation of SUVmax of different abdominopelvic regions in predicting incomplete tumor debulking and found it more predictive than the peritoneal cancer index at surgery [24]. Herein, we used the number of metabolic active peritoneal sites as a surrogate of PC distribution in predicting surgical resectability after taking consideration of other quantitative metabolic parameters of tumor burden and clinical parameters. This method could be easily incorporated into routine clinical practice without the need of more labour-intensive effort of quantitative analysis but would rely on the experience of the reading radiologist or nuclear medicine physician in the result interpretation, hence a degree of subjectivity. Nevertheless, visual assessment has been previously shown to be as robust as quantitative analysis [25–27]. This method also provided semi-quantitative measurement of disease distribution as additional information for the gyneoncologist or surgeon.

There were limitations of the present study. First, this was a retrospective study in a small-size cohort of a single center experience, therefore the results will require prospective validation in larger population and preferably multicenter study. With a larger size of population, the location of PC can be taken into account for prediction of surgical resectability. As with predictive models derived from computer tomography [28, 29] and magnetic resonance imaging [30], results from this study would likely be an early step to add into the current literature in the use of imaging predictive markers of incomplete debulking surgery. Further prospective multi-institution studies are required to validate these preliminary findings. Second, patients enrolled in the current study included all disease stages and could overstate the use of ^18^F-FDG PET/CT as choice between UDS and NAC followed by IDS is restricted to patients with advanced disease [6, 7]. However, by including all stages, we were able to show that PC was an adverse factor for complete tumor debulking and subsequent analyses support that the PC spread and tumor burden affected the immediate surgical outcome. Finally, the differences in the philosophy and approach of individual gyneoncologist or surgeon in obtaining maximal debulking were challenging to be factored into consideration.

## Conclusion

The number of metabolic active peritoneal sites derived from ^18^F-FDG PET/CT was associated with incomplete debulking surgery and potentially useful in assisting treatment stratification in patients with ovarian and peritoneal cancers.

## Data Availability

The datasets used and/or analyzed during the current study are available from the corresponding author on reasonable request.
